# HiChIP and Hi-C Protocol Optimized for Primary Murine T Cells

**DOI:** 10.3390/mps4030049

**Published:** 2021-07-16

**Authors:** Tomas Zelenka, Charalampos Spilianakis

**Affiliations:** 1 Department of Biology, University of Crete, GR70013 Heraklion, Crete, Greece; zelenkt@gmail.com; 2 Institute of Molecular Biology and Biotechnology—Foundation for Research and Technology Hellas, GR70013 Heraklion, Crete, Greece

**Keywords:** chromatin organization, HiChIP, Hi-C, 3C, nucleus, long-range interactions, T cells, thymocytes

## Abstract

The functional implications of the three-dimensional genome organization are becoming increasingly recognized. The Hi-C and HiChIP research approaches belong among the most popular choices for probing long-range chromatin interactions. A few methodical protocols have been published so far, yet their reproducibility and efficiency may vary. Most importantly, the high frequency of the dangling ends may dramatically affect the number of usable reads mapped to valid interaction pairs. Additionally, more obstacles arise from the chromatin compactness of certain investigated cell types, such as primary T cells, which due to their small and compact nuclei, impede limitations for their use in various genomic approaches. Here we systematically optimized all the major steps of the HiChIP protocol in T cells. As a result, we reduced the number of dangling ends to nearly zero and increased the proportion of long-range interaction pairs. Moreover, using three different mouse genotypes and multiple biological replicates, we demonstrated the high reproducibility of the optimized protocol. Although our primary goal was to optimize HiChIP, we also successfully applied the optimized steps to Hi-C, given their significant protocol overlap. Overall, we describe the rationale behind every optimization step, followed by a detailed protocol for both HiChIP and Hi-C experiments.

## 1. Introduction

**Figure 1:**
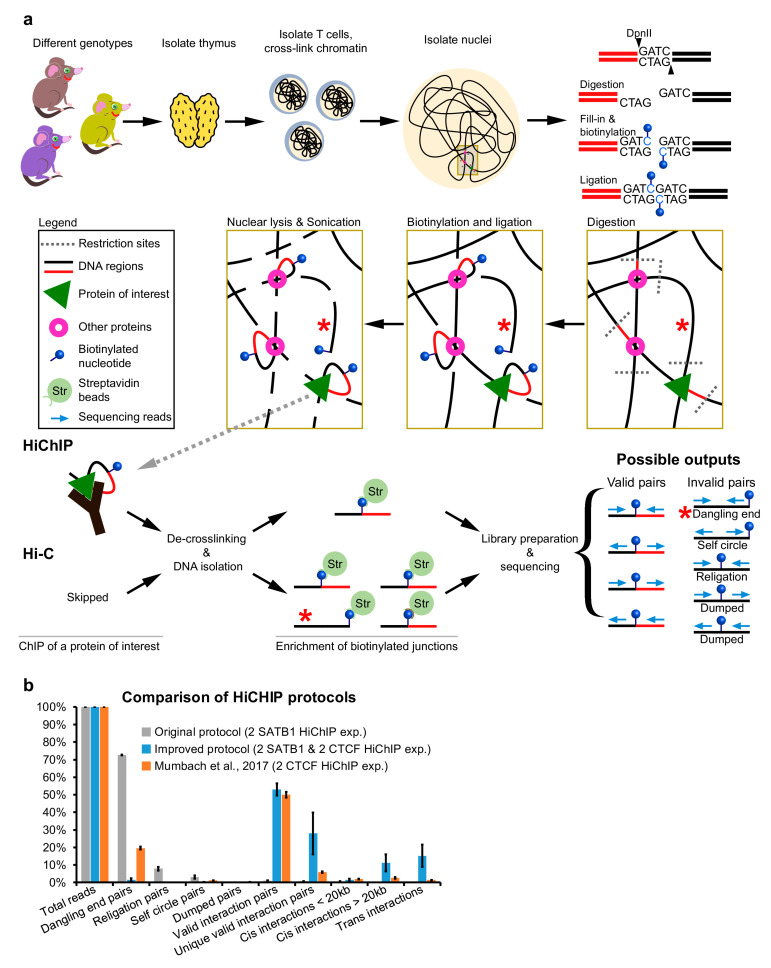
Overview of the HiChIP protocol. (**a**) Outline of the HiChIP protocol. The red star indicates fragments with dangling ends. (**b**) Comparison of the sequencing read statistics of SATB1 and CTCF HiChIP experiments. The original HiChIP protocol performed in our laboratory yielded a high number of fragments with dangling ends. The improved version of the protocol provided in this work substantially decreased the number of invalid pairs, including the dangling end pairs. Comparison with the publicly available CTCF HiChIP experiment [15] is provided.

Understanding chromatin architecture is becoming the next gold standard when studying processes inside eukaryotic cells. Since the advent of the chromosome conformation capture technique [1], there has been a vast development of methods allowing to probe long-range chromatin interactions on a genome-wide scale [2,3,4]. Such widely used research approaches are Hi-C [5], or its variants and methods that enrich for protein-mediated long-range chromatin interactions, such as ChiA-PET [6,7], PLAC-seq [8] and HiChIP [9]. In this work, we describe the key steps of an optimized HiChIP protocol that can also be used to prepare Hi-C libraries since both techniques share a significant part of the protocol steps. The whole approach is based on the in situ version of the Hi-C protocol [10], where all the initial steps take place in intact nuclei to reduce the probability of generating chimeric DNA molecules as a result of ligating DNA fragments originating from different cells. The protocol’s outline is depicted in Figure 1a. Briefly, a suspension of single cells is crosslinked, and the nuclei are isolated. Nuclear DNA is enzymatically digested, and the resulting 5′ overhanging ends are filled in with biotinylated nucleotides to blunt and label them. The resulting DNA ends are ligated together, aiming to generate chimeric ligation products between two genomic fragments that were in close spatial proximity within the cell nucleus. Then, the nuclei are lysed, and the ligated DNA is fragmented by sonication. In the case of a Hi-C experiment, these fragments are directly used to prepare a sequencing library. In a HiChIP experiment, a protein of interest is further pulled down, as in a typical ChIP-seq experiment, and then the DNA is isolated and used for a DNA library construction destined for next-generation sequencing.

Our motivation to optimize this protocol stemmed from the enormous number of fragments with dangling ends that we retrieved during our initial experimental attempts (Figure 1b). Interestingly, we realized that there were more research laboratories experiencing the same issue, even though they were using different model systems. Dangling ends represent a situation where both sequencing reads (pointing inwards) are mapped to the same restriction fragment (typically unligated; labeled with a red star in Figure 1a) [11,12]. The primary cause for the generation of dangling ends lies in the insufficient ligation reaction; however, other suboptimal steps would also contribute to their generation. If the sequencing reads map to the same restriction fragment and points outwards, it is an indication of a self-ligated circularized fragment (self circle pairs in Figure 1a). Another instance is when sequencing reads are mapped to different neighboring restriction fragments that were ligated back together (religation pairs in Figure 1a). As such, any of these instances do not provide relevant information about 3D chromatin organization and should be discarded from the bioinformatic analysis. On the other hand, in a HiChIP experiment, they can be used to infer a binding site of the immunoprecipitated protein [13]; nevertheless, this is not the reason why one would perform a highly demanding HiChIP experiment. Additionally, reads with the same orientation (mapped to the same strand) are considered errors, and they should be dumped (dumped pairs in Figure 1a). This situation is typically a result of a mapping error or random breaks.

We have optimized every step of the protocol, including the DNA library construction, by tagmentation (i.e., using the viral Tn5 enzyme) to minimize the risk of forming fragments with dangling ends and to overall improve the quality and reproducibility of the protocol. As an experimental model, we have used murine primary T cells, which are usually a difficult cell type for genomics approaches due to their compact nucleus [14]. Even research groups with an optimized HiChIP protocol, functional for other cell types, may face issues when applying it to T cells, as demonstrated by the suboptimal libraries prepared for CTCF HiChIP (Figure 1b) [15]. In the following chapters, we describe the rationale of all the optimization steps that allowed us to minimize dangling ends generation while reaching ~50% of valid interaction pairs across both Hi-C and HiChIP experiments in T cells.

## 2. Experimental Design

Both Hi-C and HiChIP experiments are performed in the same way up until the immunoprecipitation step. In this protocol, we used primary murine thymocytes, but we encourage readers to test it out also in other cell types. In HiChIP experiments, we recommend using 10 million cells to ensure sufficient yield of DNA after the immunoprecipitation step. A higher number of cells may decrease the efficiency of enzymatic reactions. For this reason, the fill-in and ligation steps were optimized for 5 million cells; hence each sample of 10 million cells was split in half. In Hi-C experiments, 5 million cells are enough as a starting point for high-quality DNA library preparation, so it is not necessary to split the sample in any step.

### 2.1. Cell Lysis and Digestion

**Figure 2:**
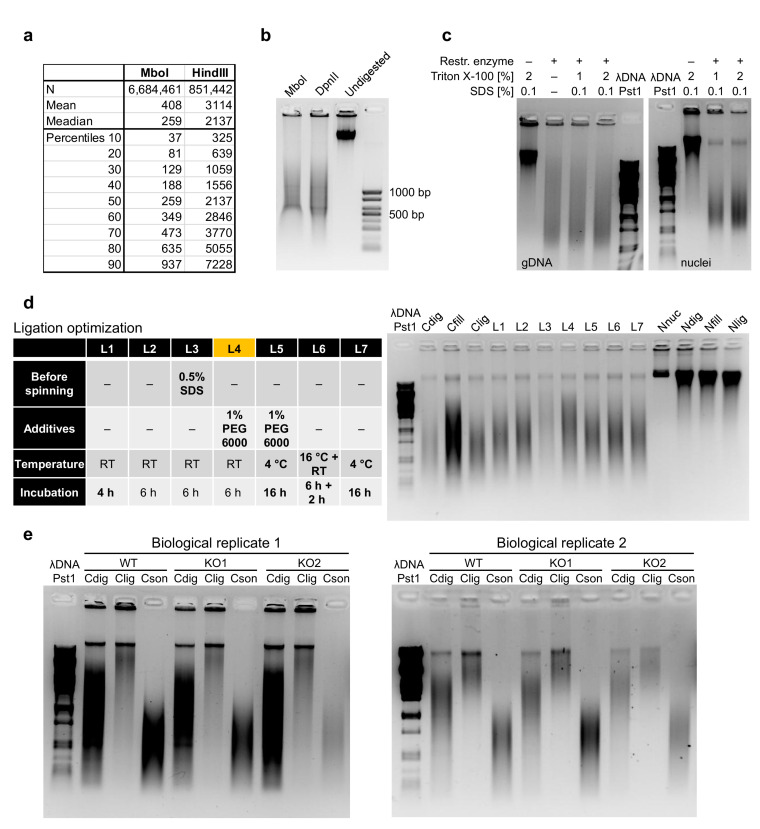
Optimization of the HiChIP protocol. (**a**) Length fragment distribution after in silico digestion of the murine genome by MboI and HindIII, as two examples of the most commonly used restriction enzymes in Hi-C and HiChIP experiments, representing a four-cutter and a six-cutter, respectively. (**b**) Comparison of murine DNA sizes upon MboI and DpnII digestion revealed no major differences in the smear size. (**c**) Examples of DpnII digestion optimization under different conditions applied on genomic DNA and on isolated thymic nuclei. The DpnII digestion buffer (NEB, B0543S) was enriched by SDS and Triton X-100 as indicated to mimic the HiChIP protocol conditions. (**d**) Comparison of selected ligation conditions revealed that condition L4 was the best performing. Three controls, Cdig, Cfill and Clig, represent samples for which enzyme in the indicated step was not added. Moreover, four controls, Nnuc, Ndig, Nfill and Nlig, were used for which no enzymes were used at any step, and samples were collected at the indicated step. These controls showed that the temperature changes and extended incubations did not affect chromatin integrity. (**e**) The optimized protocol was highly reproducible, as demonstrated by the comparison of control samples from two biological replicates from three different genotypes. Three controls provided represent the size distribution of isolated DNA after the digestion step, ligation step and after sonication, thus prior to the chromatin immunoprecipitation step.

**Figure A1:**
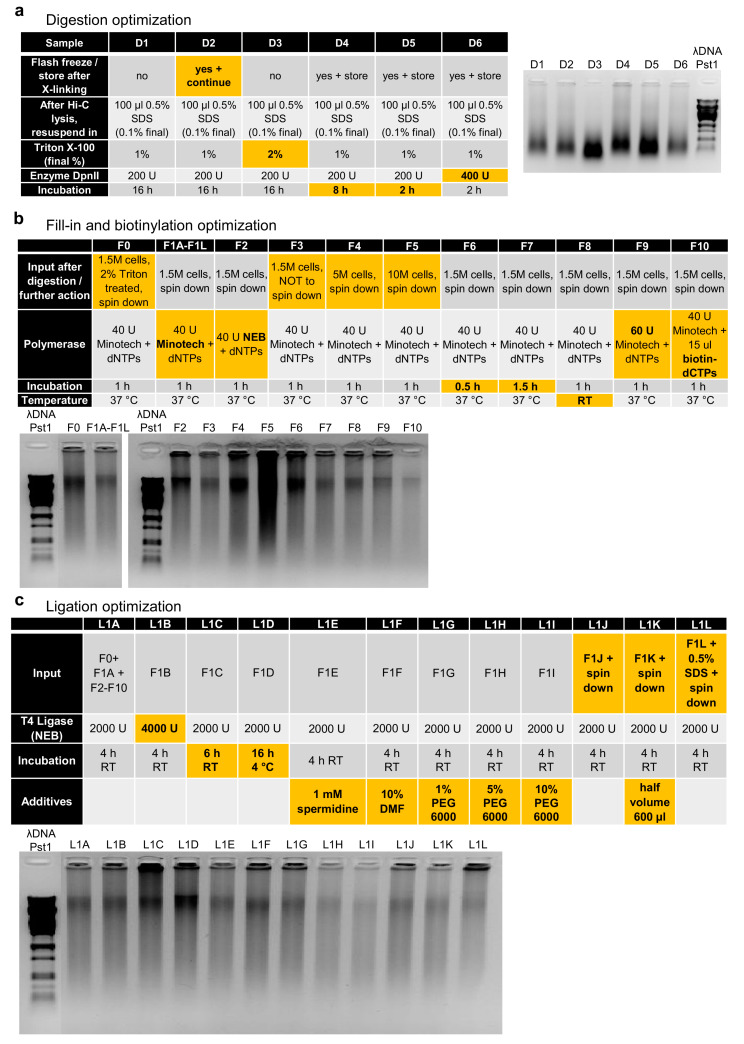
Detailed overview of optimization steps. (**a**) Optimization of the digestion step. Modified parameters are highlighted. (**b**) Optimization of the nucleotide fill-in and biotinylation step. Each of the samples F0 and F2–F10 were followed by the same ligation setup (L1A in (**c**) to estimate the impact of different fill-in parameters (highlighted). (**c**) Optimization of ligation step. The same conditions for nucleotide fill-in were applied (F1A–F1L) in (**b**) to estimate the impact of different ligation parameters (highlighted).

The prerequisite for a successful experiment is to sufficiently lyse the cell membrane followed by isolation of the crosslinked nuclei. We always monitor the success of nuclei isolation, as previously suggested [16], by checking the integrity of cell membranes upon cell lysis, with trypan blue staining and microscopic evaluation. The insufficient isolation of nuclei would decrease the efficiency of the next steps, while extended lysis could dramatically decrease the yield of intact nuclei to be analyzed. All operations with isolated nuclei must be carried out with care not to damage them. Once the nuclei are isolated, they are treated with SDS, which further increases the risk of breaking them. This treatment ensures that the nucleus and chromatin are more approachable for the restriction enzyme. The selection of the proper restriction enzyme depends on the specific research question; however, in Figure 2a, we present the DNA fragment size distribution upon in silico digestion of the murine genome, by either MboI or HindIII, as examples of four and six base-pair cutters, respectively. Although MboI was successfully used in the pilot HiChIP study [9], it is supposedly sensitive to CpG methylation, so it is recommended to use its isoschizomer DpnII [17]. Digesting genomic murine DNA side by side with these two enzymes, we could not detect any difference in the relative sizes of DNA smear (Figure 2b), but there may be some differences at a finer resolution. To optimize the step of restriction enzyme digestion, we compared different durations, amounts of restriction enzyme and the concentration of Triton X-100. Triton X-100 at this step is primarily used to quench the SDS; however, it was also shown to positively impact the efficiency of restriction enzyme digestion [18]. Indeed, the increase in Triton X-100 concentration from 1% to 2% provided better digestion results (Figure 2c and Figure A1a). This step is quite important since insufficient restriction enzyme digestion will decrease the efficiency of further steps.

### 2.2. Fill-in, Biotinylation and Ligation

In the next part of the protocol, the resulting 5′-end overhangs are filled in with nucleotides where one kind of nucleotides is biotinylated to label the end-to-end junction for the subsequent enrichment step. The length of the linker that attaches the biotin moiety to a specific nucleotide is reflected in the efficiency of its incorporation by DNA polymerase. The longer the linker, the less efficient the incorporation of the modified nucleotide is, but at the same time, the more efficient the recognition and biotin pull-down in the subsequent steps are. A 16-atom linker represents a compromise between these two aspects. In our experiments, we used biotinylated dNTPs, although biotinylated dATPs can also be used [17]. To optimize the biotinylation and ligation steps of the protocol, we performed a set of experiments with one condition being different at a time. For the optimization of the fill-in step, all the subsequent DNA ligation reactions were performed under the same conditions to track the fill-in efficiency, based on the efficiency of ligation for each experimental setup (Figure A1b). Similarly, a set of identical fill-in experiments was performed, followed by a ligation step, performed under different experimental conditions, to identify the most efficient ligation setup (Figure A1c).

To prevent any inhibitory effects of the digestion buffer and/or residual SDS on the fill-in efficiency, we spun down the nuclei and discarded all the supernatant. In the fill-in and biotinylation steps, we mainly focused on the identification of the optimal temperature, time and amount of the enzyme. The large fragment of DNA polymerase I (Klenow) is used in this reaction to generate blunt DNA ends. The goal is to maximize the reaction efficiency to overcome the hurdles of biotinylated nucleotides incorporation. However, the Klenow fragment still retains its 3′ to 5′ exonuclease activity, which competes with the polymerase activity [19] and that can, under certain conditions, even digest blunt DNA ends [20]. Given the increased amount of enzyme needed in the HiChIP experiment together with the problematic biotinylated substrate, the exonuclease activity of the enzyme needs to be considered. The mutated Klenow fragment lacking its 3′ to 5′ exonuclease activity creates a single nucleotide overhang [21], which would inhibit the ligation reaction; thus, it cannot be used either. This fact is probably reflected in our data as the best result was achieved after only 30 min of treatment while both increased time and amount of enzyme displayed worse performance (Figure A1b). Similar results could have probably been achieved in lower temperatures and extended incubation times, but we did not test them. To overcome the 3′ to 5′ exonuclease activity, we also incorporated non-heat inactivation of the Klenow enzyme by 0.5% SDS treatment. To overcome the inhibitory effect of SDS, we quenched it with Triton X-100, which also made the nuclei pellet visible upon centrifugation.

Before the ligation step, the nuclei should be again spun down and the supernatant discarded, as the presence of the residual dATP could compete with the ATP necessary for the ligation reaction [22]. For the ligation part, we again optimized the time, temperature and amount of the enzyme, but this time we also compared the effect of several additives to make sure that the blunt ends were efficiently joined. First, we tested polyethylene glycol (PEG), as a known crowding agent facilitating many enzymatic reactions and that is also used in the quick ligation kits. It has been previously shown to dramatically increase the ligation efficiency [23,24,25], depending on its concentration and molecular weight. As another crowding agent, we tested dimethylformamide (DMF), and last, we investigated spermidine, which was also shown to improve the ligation efficiency in the past [26]. The most efficient combination appeared to be either 1% PEG 6000 or 10% DMF together with a 6 h incubation at room temperature (Figure 2d and Figure A1c).

The established protocol is highly reproducible, as demonstrated by the three-control comparison (before ligation, after ligation and after sonication) from samples of three different genotypes and from two biological replicates (Figure 2e).

### 2.3. Optimization of DNA Library Preparation

The first part of the protocol is applicable for both Hi-C and HiChIP experiments, and although it is optimized for T cells, it could also be applicable for other cell types. The next steps of the HiChIP experiment, e.g., nuclear lysis, shearing and immunoprecipitation, are highly dependent on the cell type used, and they should be optimized accordingly. In this work, we provide a protocol optimized for murine T cells.

The DNA library preparation steps are again universal since they employ the manipulation of pure DNA. In this protocol, we describe the DNA library preparation by the Nextera Kit from Illumina, which is easy and convenient. To lower the costs, it can alternatively be replaced by the use of a custom-made Tn5 enzyme [27]. However, traditional methods based on adaptor ligation are also possible. In that case, we recommend performing an additional size selection step before the library construction, followed by end-repair and biotin pull-down as previously described [17].

**Figure 3:**
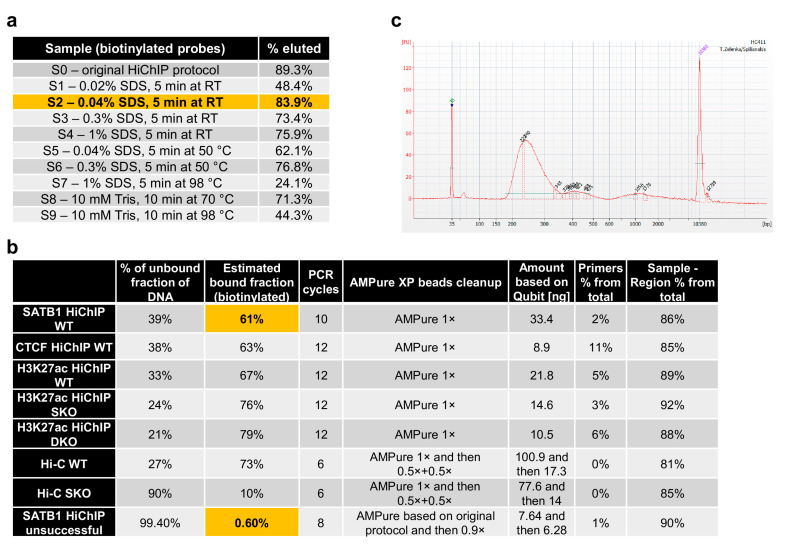
Quality control steps during sequencing library construction. (**a**) Optimization of the striping conditions to inactivate and strip away Tn5 from DNA showed that even a relatively high SDS concentration can be used because they did not affect the binding of biotinylated fragments on streptavidin beads, providing comparable results to the original HiChIP protocol. (**b**) Examples of quality control and PCR setup in the final steps of library preparation. The comparison between the original protocol and the optimized protocol indicates that the supernatant collected after binding of biotinylated DNA fragments onto streptavidin beads can be used to quantify the non-biotinylated portion of DNA to further evaluate the quality of the sequencing library. (**c**) Example of the fragment size distribution of the final sequencing HiChIP library detected by the Agilent 2100 Bioanalyzer system.

The Tn5 enzyme tends to stay tightly bound onto DNA [27,28], which would inhibit the downstream PCR amplification of DNA or at least the first “gap-filling” step required upon tagmentation. For this reason, we sought for a solution to strip away Tn5 with the unwanted fragments of DNA while keeping the biotinylated fragments bound on the streptavidin beads. We found that up to 0.3% final concentration of SDS, which is sufficient to strip away Tn5 [28], would still preserve binding of the biotinylated DNA fragments onto the streptavidin beads, providing a similar efficiency to the original HiChIP protocol, which lacks the SDS treatment (Figure 3a). Tn5 and non-biotinylated DNA fragments are then washed away, and the beads with purified ligation junctions are used for PCR amplification. The efficiency of biotinylation and theoretically even the stripping step can also be monitored by comparing the amount of input DNA and the amount of DNA in the supernatant (Figure 3b). Upon PCR amplification, we usually perform clean-up and size selection of DNA using AMPure beads. In this protocol, we provide AMPure beads to sample ratios that usually work well for us. However, this step can be optimized for each batch of beads by purifying 100 bp agarose gel ladder with different AMPure beads to ladder ratios to identify the optimal one. An example of the fragment size distribution of the working HiChIP library is provided in Figure 3c.

### 2.4. Chemicals and Enzymes

- PBS (Sigma-Aldrich, St. Louis, MO, USA, cat. no. P4417)
- 16% paraformaldehyde aqueous solution (Electron Microscopy Sciences, Hatfield, PA, USA, cat. no. 15710)
- NaCl (Merck, Kenilworth, NJ, USA, cat. no. 106404)
- EDTA (Sigma-Aldrich, St. Louis, MO, USA, cat. no. E6511)
- EGTA (Sigma-Aldrich, St. Louis, MO, USA, cat. no. E8145)
- Hepes (Sigma-Aldrich, St. Louis, MO, USA, cat. no. 54457)
- Glycine (PanReac AppliChem, Darmstadt, Germany, cat. no. A1067)
- Tris (PanReac AppliChem, Darmstadt, Germany, cat. no. A1086)
- NP-40 (Sigma-Aldrich, St. Louis, MO, USA, cat. no. 74385)
- Protease inhibitors (custom-made mixture, n/a)
- Phosphatase inhibitors (custom-made mixture, n/a)
- Trypan blue solution (Merck, Kenilworth, NJ, USA, cat. no. 93595)
- SDS (prepare 20% stock) (Sigma-Aldrich, St. Louis, MO, USA, cat. no. L5750)
- Triton X-100 (prepare 20% stock) (Merck, Kenilworth, NJ, USA, cat. no. 108603)
- PEG 6000 (prepare 30% stock) (Merck, Kenilworth, NJ, USA, cat. no. 528877)
- DpnII restriction enzyme (NEB, Ipswich, MA, USA, cat. no. R0543M)
- Biotin-16-dCTP (Jena Bioscience, Jena, Germany, cat. no. NU-809-BIO16-L)
- 10 mM dGTP (Promega, Madison, WI, USA, cat. no. U1240)
- 10 mM dTTP (Promega, Madison, WI, USA, cat. no. U1240)
- 10 mM dATP (Promega, Madison, WI, USA, cat. no. U1240)
- DNA Polymerase I, Large (Klenow) Fragment (NEB, Ipswich, MA, USA, cat. no. M0210L)
- 10× NEB T4 DNA Ligase Buffer with 10 mM ATP (NEB, Ipswich, MA, USA, cat. no. B0202)
- 20 mg/mL BSA (NEB, Ipswich, MA, USA, cat. no. B9000S)
- 400 U/μL T4 DNA Ligase (NEB, Ipswich, MA, USA, cat. no. M0202L)
- RNase A (Qiagen, Hilden, Germany, cat. no. 1007885)
- Proteinase K (Sigma-Aldrich, St. Louis, MO, USA, cat. no. P2308)
- Phenol solution (Merck, Kenilworth, NJ, USA, cat. no. P4557)
- Chloroform (Merck, Kenilworth, NJ, USA, cat. no. 102445)
- Isoamylalcohol (Merck, Kenilworth, NJ, USA, cat. no. 100979)
- Dynabeads™ Protein A (Invitrogen, Waltham, MA, USA, cat. no. 10002D)
- Dynabeads™ Protein G (Invitrogen, Waltham, MA, USA, cat. no. 10004D)
- ChIP DNA Clean and Concentrator kit (Zymo, Irvine, CA, USA, cat. no. D5205)
- Custom-made anti-SATB1 (Davids Biotechnologie, Regensburg, Germany, cat. no. n/a)
- Anti-H3K27ac (Abcam, Cambridge, United Kingdom, cat. no. ab4729)
- Anti-CTCF (Abcam, Cambridge, United Kingdom, cat. no. ab70303)
- Sodium deoxycholate (Sigma-Aldrich, St. Louis, MO, USA, cat. no. D6750)
- LiCl (Sigma-Aldrich, St. Louis, MO, USA, cat. no. L7026)
- Qubit™ dsDNA HS Assay Kit (Invitrogen, Waltham, MA, USA, cat. no. Q32851)
- Streptavidin C-1 beads (Invitrogen, Waltham, MA, USA, cat. no. 65002)
- Tween-20 (Merck, Kenilworth, NJ, USA, cat. no. 822184)
- N,N-Dimethylformamide (Sigma-Aldrich, St. Louis, MO, USA, cat. no. D4551)
- MgCl2 (Ambion, Austin, TX, USA, cat. no. AM9530G)
- Nextera DNA Sample Preparation Kit (Illumina, San Diego, CA, USA, cat. no. FC-121-1030)
- Nextera DNA Sample Preparation INDEX Kit (Illumina, San Diego, CA, USA, cat. no. FC-121-1011)
- Phusion^®^ High-Fidelity PCR Master Mix with HF Buffer (NEB, Ipswich, MA, USA, cat. no. M0531S)
- AMPure XP beads (Beckman Coulter Genomics, Chaska, MN, USA, cat. no. A63880)

## 3. Procedure

### 3.1. Cell Preparation (Required Time: 1.5 h)

1. Isolate the thymus from freshly euthanized animals and homogenize it to prepare a single-cell suspension in 10 mL of **1× PBS**. Homogenization can be achieved, for example, by rubbing the tissue by a syringe pestle against a 40 µm cell strainer (Falcon, 352340) to simultaneously separate and filter the cells.
2. Spin down the cells at 500× *g* at 4 °C for 5 min and wash them twice with 10 mL of **1× PBS**.
3. Count the cells (for example, using a hemocytometer or an automated cell counter) and use 10 million cells per HiChIP experiment or 5 million cells per Hi-C experiment. Bring up the volume to 10 mL with **1× PBS** and resuspend the cells well.
4. Add 1 mL of **Fixation Buffer** (11% Formaldehyde (methanol free), 100 mM NaCl, 1 mM EDTA, 0.5 mM EGTA and 50 mM Hepes pH 8.0).
5. Incubate the samples for 10 min at room temperature while rocking.
6. Quench the reaction by adding **Glycine** to 0.2 M final concentration (870 μL, 2.5 M stock), mix well and incubate at room temperature for 5 min.
7. Spin down the cells at 1000× *g* at 4 °C for 5 min.
8. Discard the supernatant, resuspend the cell pellet in 1 mL ice-cold **1× PBS** and transfer the solution into a fresh Eppendorf tube.
9. Spin down the cells at 1000× *g* at 4 °C for 5 min.
10. Repeat the wash (steps 8–9) one more time and discard the supernatant.

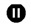
. **PAUSE STEP** Either proceed with the protocol or flash-freeze the cell pellets in liquid nitrogen (or a dry ice/ethanol bath) and store them at –80 °C. We have compared both approaches, and freezing the cells did not affect the efficiency of subsequent steps.

### 3.2. Lysis and Restriction Enzyme Digestion (Required Time: 20 h)

11. Resuspend the pellets of 10 million cells per HiChIP sample (or 5 million cells for Hi-C) in 500 μL of ice-cold **Hi-C Lysis Buffer** (10 mM Tris-HCl pH 8.0, 10 mM NaCl, 0.2% NP-40 and 1× Protease Inhibitors) and rotate them at 4 °C for 1–1.5 h.

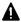
. **CRITICAL STEP** Efficient preparation of nuclei is critical, so we advise monitoring cell lysis under a microscope upon **Trypan blue** staining, which should not stain the cells with an intact cellular membrane. Extended cellular lysis up to 4 h did not negatively affect the experiment, unlike the insufficient lysis.

12. Spin down the nuclei at 2500× *g* at 4 °C for 5 min and discard the supernatant.
13. Wash the pellet once with 500 μL of ice-cold **Hi-C Lysis Buffer**.
14. Remove the supernatant and gently resuspend the pellet by pipetting up and down in 100 μL of **0.5% SDS**.
15. Incubate at 62 °C for 10 min and then add 300 μL of **ddH_2_O** and 50 μL of **20% Triton X-100** to quench the SDS.
16. Mix by pipetting and incubate at 37 °C for 15 min.
17. Add 50 μL of **10× DpnII Buffer** and check the pH with 4 μL spotted on a pH-indicator paper. If it is in the range of pH 6–7, you can proceed; otherwise, fix the pH with the addition of **Tris-HCl** pH 8.0.

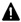
. **CRITICAL STEP** We noticed that older batches of **Triton X-100** tend to have low pH, which negatively affects the enzymatic reactions.

18. Add 4 μL (200 U) of DpnII restriction enzyme (NEB, R0543M—50 U/μL), and digest chromatin for 16 h at 37 °C, while shaking (300 rpm).
19. Heat inactivate DpnII at 62 °C, for 20 min and let it cool down to room temperature.
20. Resuspend the nuclei and split each HiChIP sample into two halves, each in a different tube. Do not split the Hi-C samples. Keep them as they are.
21. Spin down the nuclei at 2500× *g* at 4 °C for 5 min and discard the supernatant.

### 3.3. Fill-in Reaction and Proximity Ligation (Required Time: 7.5 h)

22. Resuspend the nuclei in 300 μL of **Fill-in Master Mix** according to the Table 1 below.

23. Mix by pipetting and incubate at 37 °C for 30 min while shaking (300 rpm).
24. Merge the two halves of each HiChIP sample back together (does not apply to Hi-C), mix it carefully by pipetting and keep 15 μL as the control **C1**.
25. Add 15 μL of **20% SDS** (final 0.5%) to inactivate the Klenow enzyme and mix carefully by pipetting.
26. Add 32 μL of **20% Triton X-100** (final 1%) to quench the SDS. This makes the nuclear pellet visible (translucent).
27. Incubate at 37 °C for 5 min.
28. Split the HiChIP samples into two halves again, before ligation (do not split the Hi-C sample).
29. Spin down the nuclei at 2500× *g* at 4 °C for 10 min and discard the supernatant.
30. Resuspend the nuclei in 1200 μL of **Ligation Master Mix** according to Table 2 below.

31. Incubate the reaction for 6 h at room temperature with rotation. In the meanwhile, for the HiChIP experiment, start the incubation of antibodies with the magnetic **Protein A/G DynaBeads** (needed for step 41).
32. Merge the two halves of the HiChIP samples back together (does not apply to Hi-C), mix carefully by pipetting and take 120 μL as the control **C2**.
33. Spin down the nuclei at 2400× *g* at 4 °C for 15 min and discard the supernatant.
34. To further decrease the number of dangling ends, we recommend implementing the removal of unligated, biotinylated ends [17]. For HiChIP libraries, it can either be performed right after the ligation in **T4 ligation buffer** (step 32) or after nuclei spin down (at this step, in its dedicated **T4 ligase reaction buffer)**. For the reaction, add only dGTP and dATP nucleotides and T4 DNA polymerase, which will naturally manifest its 3′-5′ exonuclease activity and remove the unligated 3′ biotin-dCTP. Alternatively, this step can be performed after DNA purification (step 67).

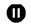
**PAUSE STEP** Either proceed with the protocol or flash-freeze the pellets of nuclei in liquid nitrogen (or dry ice/ethanol bath) and store them at –80 °C.

### 3.4. Sonication (Required Time: 1 h)

35. Resuspend the HiChIP pellets in 60 μL (and Hi-C pellets in 30 μL) of **Sonication Buffer** (1% SDS, 50 mM Tris-HCl pH 8.0, 20 mM EDTA and 1× Protease Inhibitors), pipette up and down 20 times to mix and incubate at room temperature for 15 min. Try to avoid air bubbles.
36. Add 540 μL of cold **1× TE** buffer (pH 8.0) into HiChIP samples (270 μL for Hi-C) and keep close to ice.

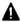
**CRITICAL STEP** SDS will start precipitating when on ice, so try to avoid it by controlling the sample and putting it on and off the ice.

37. Sonicate with a Labsonic M—Tip sonicator (or any other sonicator, based on your optimized conditions) for 1.5 min (3 cycles with 30 s ON/OFF, 40% Power). Sonication time and power may be optimized based on the cell type and instrument used.
38. Transfer the sample in a 1.5 mL Eppendorf tube and spin at 20,000× *g* at 4 °C for 20 min. Carefully collect the supernatant into a clean tube.
39. In HiChIP experiments, transfer 50 μL into a new tube as the control **C3**.
40. For solely Hi-C experiments, use the entire volume to purify the DNA in the Elution section (step 60). Alternatively, use 50 μL for Hi-C and use the rest for the HiChIP experiment.

### 3.5. HiChIP Samples—Immunoprecipitation (Required Time: 16 h)

41. For HiChIP samples, start the incubation of the magnetic **Protein A/G DynaBeads** with antibodies earlier, optimally during the ligation step (step 31).
42. Wash aliquots of 60 μL (or 30 μL + 30 μL, when bought separately) of **Protein A/G DynaBeads** for each sample, three times with 0.5 mL cold **BSA/1×PBS** (1 mg/mL BSA in 1× PBS).
43. Perform the washes as follows: Add the solution. Remove the tubes from the magnet and invert them several times to resuspend the beads. Place the tubes on the magnet and collect the beads for 2 min. Remove the supernatant. Ideally, perform all steps with the magnetic rack on ice.
44. Resuspend the beads in 1000 μL of **BSA/1×PBS + 1× Protease Inhibitors** in Protein LoBind tubes.
45. Add the antibodies of interest based on your experimental needs. The amounts range based on the type and quality of the antibody and other factors as well, so we recommend running some optimization experiments first. We used 8 μg of custom-made anti-SATB1, 2 µg of anti-H3K27ac (Abcam, Cambridge, United Kingdom, cat. no. ab4729) and 7 µg of anti-CTCF (Abcam, Cambridge, United Kingdom, cat. no. ab70303) antibodies.
46. Incubate the beads with the antibodies for 4–6 h at 4 °C with rotation.
47. Wash the antibody-coupled beads three times with 0.5 mL of ice-cold **BSA/1×PBS,** and if needed, you can leave them on ice until ready.
48. After the sonication step, add **20% Triton X-100** into each tube. Choose the volume depending on the sample volume to obtain a final concentration of 1%.
49. Mix well and incubate at 37 °C for 15 min.
50. Add an equal volume (sample + Triton) of **2× ChIP Binding Buffer** (20 mM Tris-pH 8, 2 mM EDTA, 0.2% sodium deoxycholate and 2× Protease + Phosphatase Inhibitors).
51. Combine the chromatin samples with the washed antibody-coupled beads and transfer everything into DNA LoBind tubes.
52. Incubate at 4 °C for 12–16 h with rotation.
53. **OPTIONAL STEP** You can also prepare 15 μL of **Protein A/G DynaBeads** to preclear chromatin before the immunoprecipitation step. In this case, combine washed preclearing beads with chromatin in **ChIP Binding Buffer** in DNA LoBind tubes and rotate the samples at 4 °C for 1 h. Collect the preclearing beads toward a magnet and combine the supernatant (with the precleared chromatin) with the washed antibody-coupled beads. Transfer everything into a new DNA LoBind tube and incubate at 4 °C for 12–16 h with rotation.
54. Wash the beads with the precipitated chromatin 4–7 times (depending on how much background you are getting, start with four washes) with ice-cold **RIPA Buffer** (50 mM Hepes pH 8.0, 1% NP-40, 0.70% sodium deoxycholate, 0.5 M LiCl, 1 mM EDTA and 1× Protease Inhibitors). Perform the washes as follows:
55. Place the tubes on a magnetic rack on ice. Once the beads have been collected toward the magnet, rotate the tube swiftly and let the beads travel from one side of the tube to the other. Repeat a few times, then aspirate the supernatant without disturbing the beads and repeat the washes as required.
56. Wash once with 1 mL ice-cold **TE buffer** (pH 8.0).
57. Resuspend the beads in 1 mL **TE buffer** (pH 8.0) and transfer the samples into a NEW tube.
58. Collect the beads toward the magnetic stand, discard the supernatant and continue with the next section.

### 3.6. Elution of Immune Complexes and DNA Isolation (Required Time: 20 h)

59. There are three control samples: **C1** (15 μL), **C2** (120 μL) and **C3** (50 μL) previously collected. Bring the volume up to 120 μL with **1× TE** buffer (pH 8.0); i.e., 105, 0 and 70 μL, respectively.
60. Split the Hi-C samples into two tubes, containing ~120–140 μL each. If you have lower volumes, bring it up to 120 μL with **1× TE** buffer (pH 8.0).
61. Add into each Hi-C sample, onto the HiChIP beads and also in the control samples, 125 μL of **Hi-C Elution Buffer** (10 mM Tris-HCl pH 8.0, 5 mM EDTA, 300 mM NaCl and 1% SDS), mix by pipetting and incubate at 65 °C for 6–16 h.
62. Add to HiChIP samples 110 μL of **TE Buffer** (add 115–95 μL into Hi-C samples for a total volume of 235 μL), mix by pipetting and collect the beads toward a magnetic stand. Transfer the supernatant into a fresh tube.
63. Add, into both control and HiChIP samples, 10 μL of **RNase A** (10 mg/mL) and incubate at 37 °C for 30 min.
64. Add 8 μL of **Proteinase K** (10 mg/mL stock solution) and incubate for 2–3 h at 55 °C.
65. Clean up the samples (~250 μL) using the **ChIP DNA Clean and Concentrator kit** (Zymo, D5205) or by the traditional **Phenol-Chloroform-Isoamyl alcohol** (25:24:1) method.
66. When using the kit, follow the manufacturer’s instructions. Briefly, apply five volumes of **ChIP DNA Binding Buffer** and re-load the column 2–3 times since it has a smaller capacity. Elute the DNA by applying 6 μL (12 μL for Hi-C) of pre-warmed **Elution Buffer** directly onto the center of the column matrix and incubate at room temperature for 1 minute. Spin down the tube and repeat the elution step one more time to obtain 12 μL (24 μL for Hi-C) in total.
67. Use 2 μL of each sample to quantify DNA on **Qubit** using the **dsDNA HS Assay Kit** and load 800–1000 ng of control samples on **1.5% TBE agarose gel** to qualitatively review the prepared DNA libraries (see Figure 2c).
68. Additionally, you can use a part of the **C3** control sample to set up a control PCR reaction. Ligation of two DpnII blunt DNA ends produces a novel restriction site recognized by the restriction enzyme ClaI [17]. Hence primers designed for two adjacent restriction fragments with a high likelihood to be ligated together can be used to amplify the ligated molecule. If the fragments were properly digested, filled in and re-ligated, then they should be digested by ClaI. The ratio between the digested and undigested fragments provides another piece of evidence about the protocol’s efficiency.

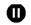
**PAUSE STEP** Purified DNA can be stored long-term at –20 °C.

### 3.7. Biotin Pull-Down and Preparation of DNA Libraries for Illumina Sequencing (Required Time: 1.5 h)

69. Use all the material left for HiChIP samples and 100 ng of Hi-C samples.
70. Bring up the volume of all the samples to 25 μL with **ddH_2_O**.
71. Prepare for biotin pull-down, for HiChIP, by washing 5 μL of **Streptavidin C-1 beads** with 500 μL of **Tween Wash Buffer** (5 mM Tris-HCl pH 7.5, 0.5 mM EDTA, 1 M NaCl and 0.05% Tween-20).
72. Resuspend the beads in 25 μL of **2× Biotin Binding Buffer** (10 mM Tris-HCl pH 7.5, 1 mM EDTA and 2M NaCl) and add them to 25 μL of DNA samples.
73. Incubate at room temperature for 20 min, resuspending from time to time by pipetting up and down.
74. Separate the beads on a magnet and transfer the supernatant into a fresh tube.
75. Use 20 μL of the supernatant to measure DNA concentration using **Qubit** as quality control.
76. Wash the beads twice with 400 μL of **Tween Wash Buffer** and resuspend them at room temperature.
77. Wash the beads with 100 μL of **1× TD Buffer** (10 mM Tris-HCl pH 7.5, 5 mM MgCl_2_ and 10% dimethylformamide).
78. Resuspend the beads in 25 μL of **2× TD Buffer**, add 20–24 μL **ddH_2_O** (depending on the amount of Tn5 used, the total volume should be 50 μL) and add the appropriate amount of **Tn5 enzyme** from the Nextera DNA Sample Preparation Kit (Illumina, FC-121-1030) based on the amount of your precipitated material.
79. Adjust the amount of Tn5 linearly for different amounts of post-ChIP DNA. Use 1 μL for the lowest yields of ~5 ng of DNA and up to 5 μL for 100 ng of DNA.
80. Incubate at 55 °C for 10 min with interval pipetting up and down to resuspend.
81. Place samples on the magnet and remove the supernatant.
82. Resuspend in 300 μL of **Strip Buffer** (0.15% SDS, 10 mM Tris-HCl pH 8.0 and 50 mM EDTA) and incubate at room temperature for 5 min to strip off and deactivate *Tn5*.
83. Wash the beads once with 400 μL of **Tween Wash Buffer.**
84. Wash the beads once with 500 μL of **10 mM Tris-HCl** (pH 8.0).

### 3.8. PCR and Post-PCR Size Selection (Required Time: 1.5 h)

85. Resuspend the beads in 50 μL of PCR master mix prepared according to Table 3. Due to different Nextera barcodes for each sample, it needs to be pipetted individually.

86. Run the PCR program according to the conditions described in Table 4 with the number of repeated cycles estimated based on the amount of post-ChIP DNA determined by Qubit. An accurate estimation of the number of PCR cycles is never an easy task. One option is to follow the recommendation from the original HiChIP paper: “Greater amount than 50 ng run for 5 cycles, approximately 50 ng for 6 cycles, 25 ng for 7 cycles, 12.5 ng for 8 cycles, etc” [9]. However, the quality of libraries is not reflected in the quantification, so this approach is really just an estimation (although often sufficient). In our laboratory, we tend to amplify a bit more, ranging between 8–14 cycles for 50–3 ng of post-ChIP DNA, respectively, in order to obtain a sufficient amount of DNA.

87. Place the amplified DNA libraries on a magnet and transfer the supernatant into new tubes.
88. Size selection should be performed based on your technical availability. In our laboratory, we use **AMPure XP beads** with success; hence, here, we describe their use.
89. Bring **AMPure XP beads** to room temperature.
90. There are usually no long DNA fragments in HiChIP libraries, so we usually perform only one-sided size selection. In some libraries, there are also long DNA fragments present. In that case, perform double-sided size selection. The ratios given here are approximate and based on our empirical experience. Each batch of beads may behave differently, so you may test out your own sample-to-bead ratios for one-sided and double-sided size selection on a 100 bp DNA ladder for agarose gels.
91. Use a 1× ratio of **AMPure XP beads** to the volume of your sample, i.e., put 50 μL of **AMPure XP beads** into 50 μL of DNA solution.
92. Mix it well on a vortex mixer or by pipetting up and down at least 10 times.
93. Incubate at room temperature for 5 min.
94. Place the tube on a magnetic rack to separate beads and remove the supernatant.
95. **OPTIONAL STEP** For double-sided size selection, first incubate with 0.5× sample volume of beads (25 μL) and keep the supernatant (discard the beads). Add to the supernatant 0.4× of the original sample volume (20 μL) of beads and incubate again. This time discard the supernatant and continue with bead wash.
96. Add 200 µL of **80%** freshly prepared **ethanol** to the tube with the beads while on the magnetic stand.
97. Incubate at room temperature for 30 s and then carefully remove and discard the supernatant.
98. Repeat the previous washing step one more time.
99. Air-dry the beads with the cap open for a maximum of ~5 min. Do not overdry the beads/DNA as it may result in lower recovery of target DNA.
100. Elute DNA from the beads by adding 15 μL of pre-warmed **Elution Buffer** (Tris-HC pH 8.0).
101. Remove the tube from the magnet. Mix it well on a vortex mixer or by pipetting up and down.
102. Incubate at room temperature for 2 min.
103. Put the tube in a magnetic rack until the solution is clear, approximately 3–5 min.
104. Transfer the supernatant to a clean DNA LoBind tube.
105. Quantify 2 μL of the DNA libraries with **Qubit** and check 1 μL on an **Agilent Bioanalyzer System**.

## 4. Expected Results

**Figure 4:**
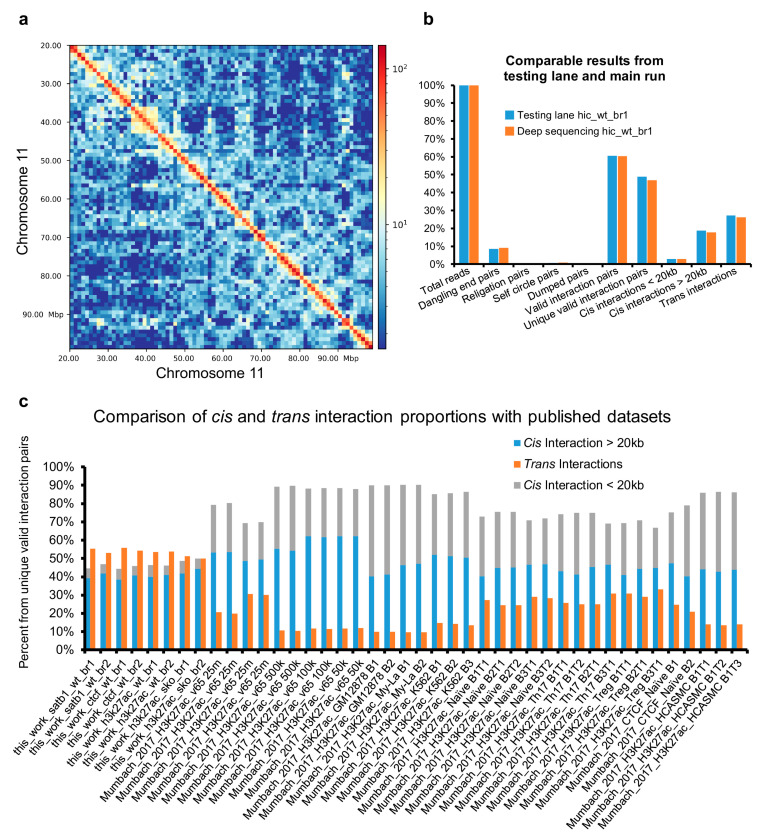
Overview of HiChIP and Hi-C data output. (**a**) Low-scale sequencing runs can aid in accurately estimating the quality of the prepared DNA libraries. Here, the Hi-C heatmap of murine thymocytes revealed the high-order chromatin organization even at a very low sequencing depth. The sample visualized had only 2.5 million total sequencing reads. (**b**) The low-scale sequencing runs highly correlated with the final deep sequencing. (**c**) The optimized protocol yielded a very low portion of short-range (<20 kbp) interactions while providing similar numbers of long-range cis and trans interactions. The comparison to HiChIP libraries produced by the group developing the original HiChIP protocol is provided.

Despite all the available controls, we recommend running a pilot low-scale sequencing experiment to evaluate the quality of libraries. In Figure 4a, we demonstrate that even as few as 2.5 million sequencing reads are enough to obtain a picture of chromosome architecture. Moreover, this small sequencing experiment provided almost identical proportions of valid interaction pairs when compared to the main sequencing experiment; thus, it can be used as a valid estimation of the library quality.

In our protocol, we usually detect a high percentage of inter-chromosomal interaction pairs (Figure 4b). In the original protocols performing the ligation step in solution, these were deemed a sign of a low-quality library as they originate from random chimeric formation between DNA fragments originating from different cells [29]. The approach used in our protocol, performing the ligation step in the nucleus, ensures the high specificity of inter-chromosomal interactions. Moreover, the frequent centrifugation steps and washes ensure that broken nuclei potentially contributing to false chimeric trans interactions would be washed away. The high percentage of inter-chromosomal interactions is likely a result of the enhanced ligation efficiency using PEG and partially due to the default compactness of the T cell nuclei. Our group has previously demonstrated the biological significance of inter-chromosomal encounters [30]; hence, it is desired not to exclude them from the analysis. The relative proportion of short and long *cis* and *trans* chromatin interactions, compared to other published studies, are depicted in Figure 4c. Apart from the high number of inter-chromosomal interactions, we would like to highlight the limited number of short-range interaction pairs (<20 kbp), which emphasizes the high efficiency of the protocol. This protocol was successfully used to investigate the 3D enhancer network of murine thymocytes using SATB1 and CTCF HiChIP experiments in wild-type cells and Hi-C and H3K27ac HiChIP experiments in both wild-type and *Satb1*^fl/fl^*Cd4*-Cre^+^ cells [31].

## 5. Reagents Setup

### 5.1. Custom-Made Protease and Phosphatase


**Inhibitor of**

**Name**

**Time**

**Stock (c)**
ProteasesAprotinin300 nM300,000ProteasesLeupeptin10 μM10,000ProteasesPepstatin A1 μM1000ProteasesEDTA10 mM500ProteasesPMSF1 mM100PhosphatasesNaF10 mM500PhosphatasesNa_3_VO_4_ (pH 10.0)2 mM200

### 5.2. Solutions

- Fixation buffer (11% formaldehyde, 100 mM NaCl, 1 mM EDTA, 0.5 mM EGTA, 50 mM Hepes pH 8.0);
- Hi-C lysis buffer (10 mM Tris-HCl pH 8, 10 mM NaCl, 0.2% NP40, 1× Protease Inhibitors);
- 0.5% SDS;
- Sonication buffer (1% SDS, 50 mM Tris-HCl pH 8, 20 mM EDTA, 1× Protease Inhibitors);
- 1× TE (pH 8);
- Hi-C elution buffer (10 mM Tris-HCl pH 8, 5 mM EDTA, 300 mM NaCl, 1% SDS);
- BSA/1×PBS (1 mg/mL BSA (0.1%) in 1× PBS);
- 2× ChIP binding buffer (20 mM Tris-HCl pH 8, 2 mM EDTA, 0.2% sodium deoxycholate, 2× Protease + Phosphatase Inhibitors);
- RIPA buffer (50 mM Hepes pH 8, 1% NP-40, 0.70% sodium deoxycholate, 0.5 M LiCl, 1 mM EDTA, 1× Protease Inhibitors);
- Tween wash buffer (5 mM Tris-HCl pH 7.5, 0.5 mM EDTA, 1 M NaCl, 0.05% Tween-20);
- 2× Biotin binding buffer (10 mM Tris-HCl pH 7.5, 1 mM EDTA, 2M NaCl);
- 1× TD buffer (10 mM Tris-HCl pH 7.5, 5 mM MgCl_2_, 10% dimethylformamide);
- 2× TD buffer (20 mM Tris-HCl pH 7.5, 10 mM MgCl_2_, 20% dimethylformamide);
- Strip buffer (0.15% SDS, 10 mM Tris-HCl pH 8, 50 mM EDTA).

## Figures and Tables

**Table 1:** Composition of the Fill-in Master Mix.

Reagent	Volume Per Reaction
10× NEBuffer 2 (NEB, M0210L)	30 μL
ddH_2_O	238.5 μL
1 mM Biotin-16-dCTP (Jena Bioscience, NU-809-BIO16-L)	15 μL
10 mM dATP (Promega, U1240)	1.5 μL
10 mM dGTP (Promega, U1240)	1.5 μL
10 mM dTTP (Promega, U1240)	1.5 μL
5 U/μL DNA Polymerase I, Klenow Fragment (NEB, M0210L)	12 μL

**Table 2:** Composition of the Ligation Master Mix.

Reagent	Volume Per Reaction
10× NEB T4 DNA Ligase Buffer with 10 mM ATP (NEB, B0202)	120 μL
20% Triton X-100 (1% final)	60 μL
2% (20 mg/mL) BSA (NEB, B9000S)	6 μL
30% PEG 6000 (1% final)	40 μL
400 U/μL T4 DNA Ligase (NEB, M0202L)	5 μL
ddH_2_O	969 μL

**Table 3:** Composition of the PCR Master Mix.

Reagent	Volume Per Reaction
Phusion HF 2× PCR Master Mix (NEB, M0531S)	25 μL
Nextera Index 1 N7XX	1–1.5 μL
Nextera Index 2 N5XX	1–1.5 μL
ddH_2_O	22–23 μL

**Table 4:** Cycling conditions for PCR.

Reaction	Temperature	Time	Cycle Number
Gap-filling required upon tagmentation	72 °C	5 min	1 cycle
Initial Denaturation	98 °C	1 min	1 cycle
Denaturation	98 °C	15 s	5–15 cycles
Annealing	63 °C	35 s	
Extension	72 °C	1 min	

## Data Availability

Any data set will be provided upon a reasonable request.
